# Evaluating the link between immune characteristics and attention deficit hyperactivity disorder through a bi-directional Mendelian randomization study

**DOI:** 10.3389/fimmu.2024.1367418

**Published:** 2024-06-05

**Authors:** Hu Jue, Chen Dan-fei, Li Fang-fang, Yu Ke-pin, Xu Jia-ye, Zhang Hui-ting, Xuan Xiao-bo, Chen Jian

**Affiliations:** ^1^ First Clinical School, Zhejiang Chinese Medical University, Hangzhou, China; ^2^ Department of Pediatrics, The Third Affiliated Hospital of Zhejiang Chinese Medical University, Hangzhou, China; ^3^ Department of Pediatrics, Zhejiang Provincial Hospital of Traditional Chinese Medicine, Hangzhou, China

**Keywords:** attention deficit hyperactivity disorder, immunophenotypes, immune cells, pleiotropy, Mendelian Randomization

## Abstract

**Context:**

Despite the recognition of attention deficit hyperactivity disorder (ADHD) as a multifaceted neurodevelopmental disorder, its core causes are still ambiguous. The objective of this study was to explore if the traits of circulating immune cells contribute causally to susceptibility to ADHD.

**Methods:**

By employing a unified GWAS summary data covering 731 immune traits from the GWAS Catalog (accession numbers from GCST0001391 to GCST0002121), our analysis focused on the flow cytometry of lymphocyte clusters, encompassing 3,757 Sardinians, to identify genetically expected immune cells. Furthermore, we obtained summarized GWAS statistics from the Psychiatric Genomics Consortium to evaluate the genetic forecasting of ADHD. The studies employed ADHD2019 (20,183 cases and 35,191 controls from the 2019 GWAS ADHD dataset) and ADHD2022 (38,691 cases and 275,986 controls from the 2022 GWAS ADHD dataset). Through the examination of genome-wide association signals, we identified shared genetic variances between circulating immune cells and ADHD, employing the comprehensive ADHD2022 dataset. We primarily utilized inverse variance weighted (IVW) and weighted median methods in our Mendelian randomization research and sensitivity assessments to evaluate diversity and pleiotropy.

**Results:**

After adjusting for false discovery rate (FDR), three distinct immunophenotypes were identified as associated with the risk of ADHD: CD33 in Im MDSC (OR=1.03, CI: 1.01~1.04, *P*=3.04×10^−5^, *P_FDR_* =0.015), CD8^br^ NKT %T cell (OR=1.08, 95%CI: 1.04~1.12, *P*=9.33×10^−5^, *P_FDR_* =0.023), and CD8^br^ NKT %lymphocyte (OR=1.08, 95%CI: 1.03~1.12, *P*=3.59×10^−4^, *P_FDR_* =0.066). Furthermore, ADHD showed no statistical effects on immunophenotypes. It’s worth noting that 20 phenotypes exist where ADHD’s appearance could diminish 85% of immune cells, including FSC-A in myeloid DC (β= -0.278, 95% CI: 0.616~0.931, *P*=0.008), CD3 in CD45RA- CD4+ (β= -0.233, 95% CI: 0.654~0.960, *P*=0.017), CD62L- monocyte AC (β=0.227, 95% CI: 0.038~1.518, *P*=0.019), CD33 in CD33^br^ HLA DR+ CD14dim (β= -0.331, 95% CI: 0.543~0.950, *P*=0.020), and CD25 in CD39+ resting Treg (β=0.226, 95% CI: 1.522, *P*=0.022), and FSC-A in monocytes (β= -0.255, 95% CI: 0.621~0.967, *P*=0.234), among others.

**Conclusion:**

Studies indicate that the immune system’s response influences the emergence of ADHD. The findings greatly improve our understanding of the interplay between immune responses and ADHD risk, aiding in the development of treatment strategies from an immunological perspective.

## Introduction

Attention Deficit Hyperactivity Disorder (ADHD), a prevalent neurodevelopmental condition among school-aged children, primarily manifests as persistent distractibility, hyperactivity, and impulsive behaviors (1), occurring in 5% of cases, with an estimated yearly worldwide impact of 491,500 disability-adjusted life years (1, 2). The diagnosis of Clinical ADHD complies with the guidelines set by the Diagnostic and Statistical Manual of Mental Disorders–5 Task Force (3). ADHD’s complex pathophysiology indicates a multifaceted interaction of genetic and environmental elements influencing neurobiological activities (4). The growing evidence accentuates immunological processes as an emerging focal point within ADHD pathophysiology, presenting a potential supplementary biological mechanism (5). Presently, the role of immune responses in the development of ADHD can be divided into three primary domains: genetic research, studies exploring links between ADHD and immune-related illnesses, and cytokine research (5). Significantly, genes linked to autoimmune disorders, such as presence of human leukocyte antigen (HLA)-DR4, HLA-DRB, and complement C4B, as being associated with an increased risk of developing ADHD (6). Disorders in neurodevelopment, such as ADHD, are marked by a reduction in the compensatory immunoregulatory system (CIRS) (z-complex of IL-4, IL-10, sIL-1RA, and sIL-2R), heightened interleukin (IL)-1 signaling linked to increased IL-1α and decreased IL-1 receptor antagonists, heightened neurogenesis, the polarization of M1/M2 macrophages, and elevated IL-4 and CC motif chemokine ligand 2 (CCL2) levels) (7). Another research (8) exploring gene activity in peripheral blood mononuclear cells revealed that in adults with ADHD, genes with varied expression levels were more prevalent in pathways linked to immune and inflammatory reactions, including the varied expression of immune genes like TNFSF8, IL7R, and C1qA. Earlier comprehensive studies have linked ADHD to autoimmune and atopic conditions, including atopic dermatitis, asthma, and allergies (9, 10). The link between the brain and the peripheral immune system via the lymphatic system is remarkable (11, 12). Research using a case-control approach indicated a rise in Treg cells, correlating with a heightened risk of ADHD (13). Research indicated that individuals diagnosed with ADHD showed notably elevated levels of CD3+ CD4+ CD25+ Foxp3+ (Tregs) in contrast to healthy individuals (8.23 ± 2.09 *vs*. 6.61 ± 2.89; z = 2.965, *p* = 0.004) (13). Earlier research has linked both CD4+ helper and CD8+ cytotoxic T cells to the development and operation of the brain (11). CD4+ T cells play a role in the development of memory, whereas the invasion of CD8+ T cells into the central nervous system interrupts the balance of microglial and neuronal functions (11, 14). This type of infiltration frequently occurs in persistent inflammatory conditions, like auto-immune and atopic diseases (15). The adaptive immune system includes CD4+ and CD8+ T cells, in addition to B cells (16). The evolution of these cells occurs from naive to central memory and effector memory cells (16, 17). CD4+ memory cells, such as Th1 (related to auto-immunity) and Th2 and Th17 (related to allergic conditions), underscore the complex connection between B and T cells, as outlined earlier (16). Although there’s a steady link between chronic immune disorders and issues with focus, our understanding of the immunological underpinnings remains scant. Up to this point, immune research has been limited to minor cytokine studies, neglecting several complicating elements in child growth that could affect the link between immune responses and issues with focus. Furthermore, the simultaneous occurrence of psychopathology remains unaddressed, casting doubt on whether its links are exclusive to attentional challenges or have a wider impact on mental health concerns. Gaining a more profound insight into the possible role of neuro-immunology in attentional issues might steer upcoming studies, improve our grasp of how attention problems develop, and aid in formulating treatment strategies.

The substantial heritability rate of 74% associated with ADHD has catalyzed extensive investigations into identifying ADHD susceptibility genes (2, 18, 19). GWAS (Genome-wide association study) methodologies facilitate the global examination of DNA variations to pinpoint links with any gene associated with ADHD. A new meta-analysis of GWAS recently pinpointed 27 key genetic sites containing DNA variations linked to a heightened likelihood of ADHD (18). Progress in extensive GWAS and Mendelian randomization (MR) methods aid in evaluating the causal links between immune characteristics and the risk of ADHD (18, 20). In the results of a study (21) of approximately 50 studies that used MR to examine causal associations with ADHD as an exposure or outcome, it was found that MR had an advantage over traditional observational designs in examining evidence of causality in ADHD, which may have preventive and therapeutic implications. Prior research has firmly confirmed the effectiveness of MR studies in investigating causal links in autoimmune disorders, assisting in circumventing confounding elements and unraveling inverse causal connections in causal deductions (22–24). Employing a two-sample MR methodology diminishes the chances of incorrect positive outcomes owing to its minimal bias towards the null hypothesis, and concurrently broadens the range of MR research (25, 26).

This research used a two-way, two-sample MR analysis to elucidate the connections between various immunophenotypes and ADHD. This methodology enables a more accurate assessment of how these immune phenotypes might impact the risk and development of ADHD, as well as whether ADHD influences the immune phenotypes. These insights offer valuable information regarding potential therapeutic targets and strategies.

## Materials and methods

### Design of the study

Employing a dual-sample Mendelian randomization approach, we assessed the bidirectional causal connections between a broad spectrum of immune cells (731 species in seven panels) and ADHD risk, drawing on extensive GWAS data. Each study integrated into the applied data was sanctioned by the relevant institutional review panels.

### Sources of GWAS data

From the GWAS Catalog, a collection of GWAS summary statistics covering 731 immune traits was gathered, with accession numbers spanning from GCST0001391 to GCST0002121 (20). The study pinpointed 122 significant independent association signals at 70 sites, 53 of which were novel, identifying the molecules and processes that control 459 cellular patterns. The study analyzed 118 total cell counts (AC), 389 median fluorescence intensities (MFI) signaling surface antigen levels, 32 morphological factors (MP), and 192 relative cell counts (RC) via flow cytometry. Within a typical group of 3757 Sardinians, 731 varied immunotypes were examined. Sample genotyping utilized four Illumina arrays (OmniExpress, ImmunoChip, Cardio-MetaboChip, and ExomeChip), employing genome-wide imputation based on a reference panel of 3514 Sardinian sequence individuals. In conclusion, the association study maintains around 22 million advanced markers, adjusted after considering gender and age as covariates (27).

Demontis and the team carried out a GWAS in 2019, concentrating on ADHD (ADHD2019) (28), and expanded their participant count in 2022 (ADHD2022) (18). The condensed result of the GWAS for ADHD2019 included 3 instances from 2018 and 35191 control participants (28). Around 210,000 rsIDs were missing from the ADHD2019 dataset. Most of these missing rsIDs were augmented with reference datasets. The comprehensive result of the GWAS on ADHD2022 included 38691 instances and 275986 control participants (18). Information regarding both ADHD instances was obtained from the Psychiatric Genomics Consortium (https://pgc.unc.edu/). Each participant originated from a European background. ADHD cases were diagnosed by psychiatrists according to ICD-10 criteria (specifically, F90.0, F90.1, and F98.8 diagnostic codes) or were individuals who had received medication tailored to ADHD symptoms. Given that a significant proportion of the GWAS participants were children, and considering the frequent co-occurrence of ADHD with other neurodevelopmental and psychiatric disorders, the study also delineated the polygenic structure of ADHD and its intersection with other phenotypes through bivariate causal mixed modeling. Additionally, polygenic scoring (PGS) analyses were conducted to investigate associations of ADHD-PGS with neurocognitive measures in the Philadelphia Neurodevelopmental Cohort (PNC). The study revealed that approximately 7.2 K (standardized = 324) common variants explained 90% of the h^2^_SNP_, thus refining the genetic architecture of ADHD. Moreover, no disparities in h^2^_SNP_ were noted between males and females in this investigation. Overall, we leveraged the largest ADHD GWAS database to date, identifying reliable variants and achieving fine localization of 27 significant loci (18).

### Choosing instrumental variables

For effective IVs, three prerequisites must be satisfied: the assumption of relevance: linking IVs to exposure; the presumption of independence: ensuring IVs are unaffected by confounding elements; and the presumption of exclusion limitation: ensuring IVs maintain conditional independence from the outcome based on the exposure. Building on earlier research (18, 20, 29), independent and notable SNPs for each immune characteristic were identified using PLINK software’s (version v1.90) clumping method (22), with a significance threshold established at 1 × 10^-5^. The threshold for linkage disequilibrium [LD] r^2^ was established at less than 0.1 within a 500 kb range, with LD r^2^ determined using the 1000 Genomes Projects reference panel (30). In the case of ADHD, the threshold for statistical significance was modified to 5×10^−6^ (clump=10000kb, r^2 =^ 0.001). In case of effector allele frequency (EAF) deletion, we queried the NCBI (https://www.ncbi.nlm.nih.gov/gds) or PhenoScanner (http://www.phenoscanner.medschl.cam.ac.uk/) databases for the EAF of SNPs, or imputation by programming. Furthermore, to eliminate the possibility of indicative pleiotropy, separate IVs of immune characteristics were extracted for additional detailed analysis. In particular, additional SNP screening was conducted to pinpoint key associations, omitting SNPs listed in the GWAS catalog and identified in this research as linked to different immune characteristics. To encompass SNPs solely linked to the outcome via exposure, where genetic differences were not connected to any factors influencing ADHD or those affecting the exposure-outcome sequence, we examined the phenotypic sign. Extensively linked to SNPs via the PhenoScanner database, this study aimed to ascertain if these phenotypes were confounders of ADHD, based on earlier MR research. Should the answer be affirmative, the implicated SNPs were eliminated. For assessing the strength of the extracted IVs, we determined the percentage of phenotypic variation explained (PVE) and the F statistic for each IV to prevent minor instrumental bias. The calculation of the F statistic utilized the equation: F = (N-K-1)/K × [R^2^/(1- R^2^)], with N representing the sample size, K the count of SNPs, and R^2^ the extent to which SNPs account for exposure. As the quantity of SNPs grows, the magnitude of the F-statistic diminishes, and conversely, as the sample size expands and the extent to which SNPs account for exposure intensifies. An F value greater than 10 is deemed a robust instrumental variable, whereas an F less than 10 is regarded as feeble. IVs exhibiting low F statistics (F < 10) were excluded from our study. Our research pinpointed a median of 18 independent instrumental variables (ranging from a minimum of 4 to a maximum of 169) linked with the 731 immunophenotypes, accounting for an average of 2.028% (spanning 0.533 to 34.123%) of the differences in their immune characteristics. Ultimately, 23 instrumental variables for ADHD were pinpointed for additional reverse-direction MR analysis.

### Mendelian randomization

For all analyses, R 4.3.1 (http://www.Rproject.org) was conducted. To detect potential anomalies in instrumental variables (31), we employed Cook’s distance, a widely used technique in regression analysis for identifying outlier metrics. A two-sample MR analysis was conducted (32) to delve into the extensive links between 731 immunophenotypes and the risk of ADHD. The primary methods for calculating causal effect sizes included inverse variance weighted (IVW) (33), weighted median-based (34), mode-based methods (35), MR pleiotropy residual sum and outlier (MR-PRESSO) (36), MR-Robust Adjustment Profile Scores (RAPS) (37), MR-Debiased IVW (38), MRLasso (39). In cases where the estimated effect varied, the standard fixed-effects IVW was substituted with the random-effects IVW approach. The diversity in the magnitude of SNP-specific causal impacts in two-sample MR was analyzed through Cochran’s Q-test (33). The MR-Egger technique was meticulously employed to detect any horizontal pleiotropy. A notable MR-Egger intercept implies that the outcomes of associations could be affected by the horizontal pleiotropic impacts of other characteristics (40). Additionally, the MR-PRESSO global test, known for its superior statistical strength, was employed to identify outliers, thereby further ruling out potential horizontal pleiotropy (36).

## Result

### Investigating how immunophenotypes impact the risk of ADHD

**Figure 1:**
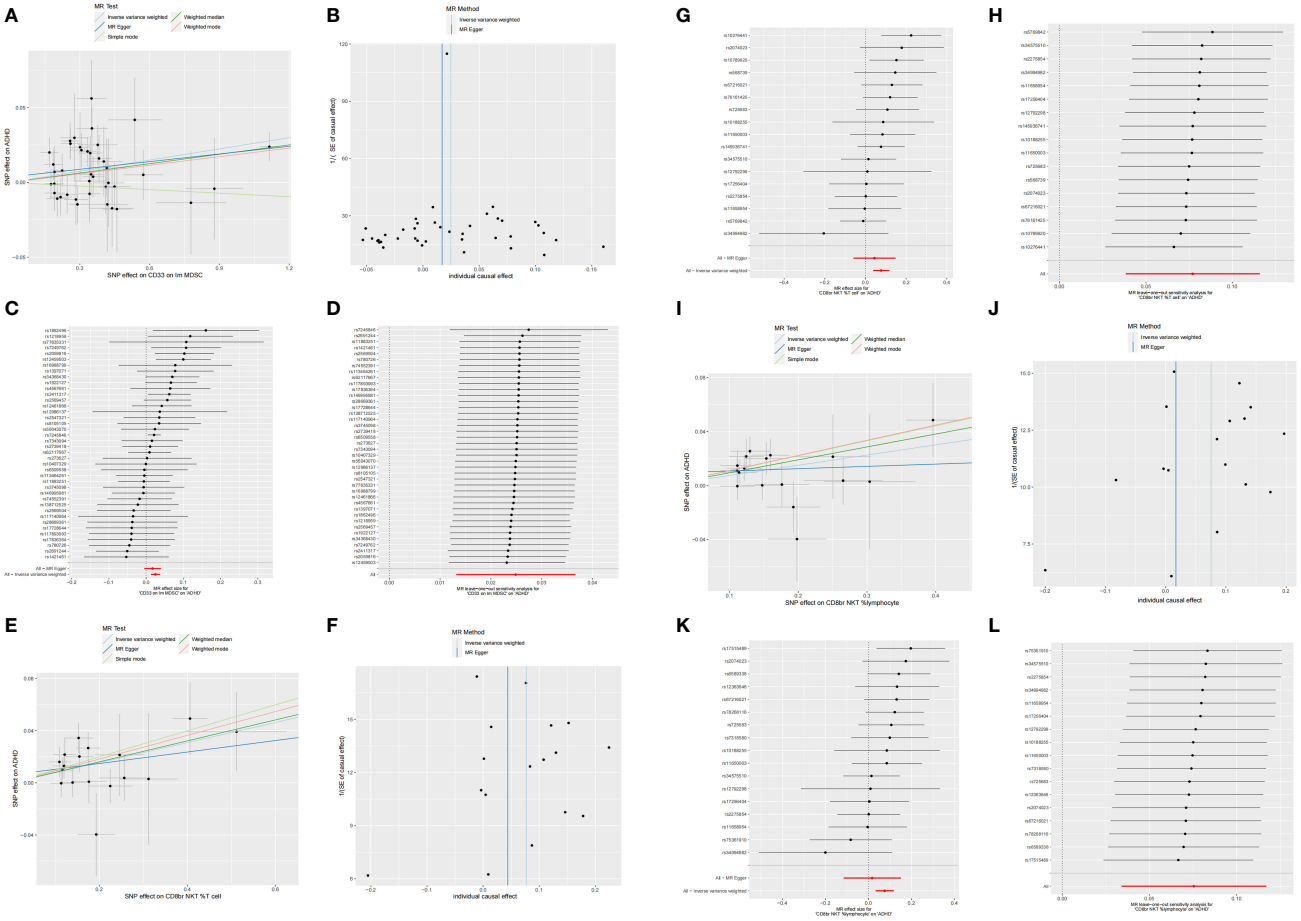
Scatter, funnel, forest, and leave one out plots the informal links between three recognized immune cells and the risk of ADHD. On the scatter plot, the x-axis represents the magnitude of the SNP’s impact due to exposure, while the y-axis indicates the extent of the SNP’s influence on the outcome. In the graphical representation, IVW estimates are denoted by blue lines, Weighted Median estimates by dark green lines, MR Egger estimates by dark blue lines, Weighted mode estimates by red lines, and simple mode estimates by pale green. **(A)** Using MR techniques, scatterplots demonstrate genetic linkages between CD33 on Im MDSC and the risk for ADHD. **(B)** Funnel plot for MR analysis with CD33 on Im MDSC as exposure and risk for ADHD as the outcome. **(C)** The forest plots illustrated the genetic links between CD33 on Im MDSC and the risk for ADHD. **(D)** The leave-one-out approach used to examine the association between CD33 on Im MDSC as the independent variable and the potential for ADHD as the dependent variable. **(E)** Using MR techniques, scatterplots demonstrate genetic linkages between CD8br NKT %T cell and the risk for ADHD. **(F)** Funnel plot for MR analysis with CD8br NKT %T cell as exposure and risk for ADHD as the outcome. **(G)** The forest plots illustrated the genetic links between CD8br NKT %T cell and the risk for ADHD. **(H)** The leave-one-out approach used to examine the association between CD8br NKT %T cell as the independent variable and the potential for ADHD as the dependent variable. **(I)** Using MR techniques, scatterplots demonstrate genetic linkages between CD8br NKT %lymphocyte and the risk for ADHD. **(J)** Funnel plot for MR analysis with CD8br NKT %lymphocyte as exposure and risk for ADHD as the outcome. **(K)** The forest plots illustrated the genetic links between CD8br NKT %lymphocyte and the risk for ADHD. **(L)** The leave-one-out approach used to examine the association between CD8br NKT %lymphocyte as the independent variable and the potential for ADHD as the dependent variable.

We identified 68 immune cells playing a causative role in ADHD at a minimal level of significance. Elevated counts of 53 immune cells and reduced counts of 15 immune cells may lead to a heightened risk of ADHD. The distribution of these 68 immune cells is as follows: 12 in B cell, 5 in cDC, 6 in Maturation stages of T cell, 3 in Monocyte, 15 in Myeloid cell, 10 in TBNK, and 17 in Treg panels (**Supplementary Table S1**). Post adjusting for multiple tests using the FDR technique, a reduced number of immunophenotypes were detected, with a significance level of 0.05. With a significance level of 0.10 (*P_FDR_* <0.1) (22), four immunophenotypes showed impacts on ADHD: CD33 on Im MDSC (Myeloid cell), CD8^br^ NKT %T cell (TBNK), CD8^br^ NKT %lymphocyte (TBNK), and CD33 on CD14+ monocyte (Myeloid). The MR-Egger regression analysis reveals that the intercept term’s p-value for CD33 on CD14+ monocytes exceeds 0.05, indicating genetic pleiotropy between SNPs and ADHD (*P* = 0.033, **Supplementary Table S2**). Consequently, the subsequent text will concentrate solely on the analysis of the initial three immunophenotypes. Comprehensive information regarding the trio of instrumental variables is detailed in **Supplementary Table S3**. Lacking horizontal pleiotropy in IVs, IVW emerged as the main technique for determining the causal link between genetic predisposition to immunophenotypic traits and a heightened ADHD risk. The estimated OR for CD33 on Im MDSC regarding ADHD risk stood at 1.03 (IVW result: 95% CI: 1.01~1.04, *P*=3.04×10^−5^, *P_FDR_* = 0.015). Results of the other methods included: weighted mode, OR=1.02, CI: 1.00~1.04, *P* = 0.032; weighted median, OR=1.02, CI: 1.00~1.04, *P*=0.011 (**Table 1** and **Supplementary Table S2**). And MR-Egger regression intercept value of 0.004, suggesting an absence of genetic pleiotropy (*P* = 0.414). The MR-PRESSO test (β = 0.025, SD = 0.006, *P*=1.64×10^−4^) and the MR-PRESSO global test revealed an absence of genetic pleiotropy bias or anomalies (*P* = 0.892, **Supplementary Table S4**). The OR for the impact of CD8^br^ NKT %T cell on the risk of ADHD was calculated to be 1.08 (IVW result: 95% CI: 1.04~1.12, *P*=9.33×10^−5^, *P_FDR_* = 0.023). Results of the other method included: weighted median, OR=1.08, 95% CI: 1.02~1.15, *P*=0.005 (**Table 1** and **Supplementary Table S2**). MR-PRESSO (β = 0.077, SD = 0.020, *P*=1.25×10^−3^), and the MR-PRESSO global test revealed an absence of genetic pleiotropy bias or anomalies (*P* = 0.458, **Supplementary Table S** **4**). The OR for the impact of CD8^br^ NKT %lymphocyte on the risk of ADHD was approximated at 1.08 (IVW result: 95% CI: 1.03~1.12, *P*=3.59×10−4, *P_FDR_* = 0.066). Results of the other method included: weighted mode (OR=1.12, 95% CI: 1.01~1.23, *P*=0.039); weighted median, OR=1.10, 95% CI: 1.04~1.16, *P*=1.00×10^−3^ (**Table 1** and **Supplementary Table S** **2**), MR-PRESSO (β = 0.075, SD = 0.020, *P*=1.79×10^−3^), and the MR-PRESSO global assessment, which revealed an absence of genetic pleiotropy bias or anomalies (*P* = 0.054, **Supplementary Table S** **4**). In-depth data derived from the sensitivity analysis confirmed the solidity of the observed causal links (**Supplementary Table S2**). The steadiness of the outcomes is also evidenced by scatter plots, funnel plots, and leave one out plots (**Figure 1**).

**Table 1:** The forest plots illustrated the causal links between ADHD and characteristics of immune cells.

immune cell	nsnp	Method	OR (95% CI)	pval	FDR
CD8br NKT %T cell	17	IVW	1.08 (1.04-1.12)	9.33e-05*	0.023
	17	Weighted median	1.08 (1.02-1.15)	0.005*	
	17	MR Egger	1.04 (0.94-1.16)	0.415	
	17	Simple mode	1.10 (1.00-1.22)	0.072	
	17	Weighted mode	1.10 (1.00-1.20)	0.075	
CD8br NKT %lymphocyte	17	IVW	1.08 (1.03-1.12)	3.59e-04*	0.066
	17	Weighted median	1.10 (1.04-1.16)	1.00e-03*	
	17	MR Egger	1.02 (0.89-1.16)	0.804	
	17	Simple mode	1.12 (1.01-1.24)	0.050	
	17	Weighted mode	1.12 (1.01-1.23)	0.039*	
CD33 on CD14+ monocyte	59	IVW	1.02 (1.01-1.03)	4.04e-05*	0.015
	59	Weighted median	1.02 (1.00-1.03)	0.022*	
	59	MR Egger	1.00 (0.99-1.02)	0.660	
	59	Simple mode	1.01 (0.97-1.05)	0.654	
	59	Weighted mode	1.02 (1.00-1.03)	0.027*	
CD33 on Im MDSC	40	IVW	1.03 (1.01-1.04)	3.04e-05*	0.015
	40	Weighted median	1.02 (1.00-1.04)	0.011*	
	40	MR Egger	1.02 (1.00-1.04)	0.135	
	40	Simple mode	0.99 (0.95-1.04)	0.717	
	40	Weighted mode	1.02 (1.00-1.04)	0.032*	

### Investigating how the emergence of ADHD influences immunophenotypes

Investigating the impact of ADHD development on immune responses, we conducted an MR analysis to understand ADHD’s causal influence on immune cells. Post-adjustment for various tests, a causal link that achieved the FDR significance level of 0.05 was not identified. At a minimal significance threshold, ADHD’s impact was observed on 20 immune cells, with the emergence of ADHD potentially reducing 17 immune cells and elevating 3 (**Table 2** and **Supplementary Table S** **5**). The distribution of these 20 immune cells occurs across various cell types: cDC (6 cells), Maturation stages of T cell (3 cells), Myeloid cell (3 cells), and Treg panels (8 cells). This includes FSC-A on myeloid DC (β= -0.278, 95% CI: 0.616~0.931, *P*=0.008), CD3 on CD45RA-CD4+ (β= -0.233, 95% CI: 0.654~0.960, *P*=0.017), CD62L-monocyte AC (β=0.227, 95% CI: 0.038~1.518, *P*=0.019), CD33 on CD33^br^ HLA DR+ CD14dim (β= -0.331, 95% CI: 0.543~0.950, *P*=0.020), and CD25 on CD39+ resting Treg (β=0.226, 95% CI: 1.033~1.022, *P*=0.022), and FSC-A on monocyte (β= -0.255, 95% CI: 0.21~0.67, *P*=0.234), among others (**Table 2** and **Supplementary Table S6**).

**Table 2:** IVW findings on how ADHD causally impacts the characteristics of immune cells.

immune cell	nsnp	OR (95% CI)	p-unadj	beta	se	Panel
CD62L- monocyte AC	23	1.25 (0.04-1.52)	0.019*	0.23	0.10	cDc
CD28- CD127- CD25++ CD8br %CD8br	23	1.21 (1.00-1.45)	0.047*	0.19	0.09	Treg
CD3 on CD45RA-CD4+	23	0.79 (0.65-0.96)	0.017*	-0.23	0.10	Maturation stages of T cell
CD3 on CM CD8br	23	0.82 (0.68-1.00)	0.044*	-0.20	0.10	Maturation stages of T cell
CD3 on activated Treg	23	0.82 (0.67-0.99)	0.038*	-0.20	0.10	Treg
CD3 on secreting Treg	23	0.82 (0.68-1.00)	0.049*	-0.19	0.10	Treg
CD3 on activated & secreting Treg	23	0.81 (0.67-0.99)	0.035*	-0.21	0.10	Treg
CD3 on CD28+ CD4+	23	0.81 (0.67-0.98)	0.030*	-0.21	0.10	Treg
CD3 on CD28+ CD45RA- CD8br	23	0.83 (0.69-1.00)	0.049*	-0.19	0.10	Treg
CD3 on CD4+	23	0.81 (0.67-0.98)	0.026*	-0.21	0.10	Treg
CD25 on CD39+ resting Treg	23	1.25 (1.03-1.52)	0.022*	0.23	0.10	Treg
CD123 on plasmacytoid DC	23	0.81 (0.67-1.00)	0.042*	-0.20	0.10	cDC
CD123 on CD62L+ plasmacytoid DC	23	0.82 (0.67-1.00)	0.043*	-0.20	0.10	cDC
CD33 on CD33br HLA DR+CD14dim	23	0.72 (0.54-0.95)	0.020*	-0.33	0.14	Myeloid cell
CD33 on CD33br HLA DR+	23	0.73 (0.55-0.98)	0.033*	-0.31	0.15	Myeloid cell
CD33 on CD33br HLA DR+ CD14-	23	0.73 (0.54-0.98)	0.036*	-0.32	0.15	Myeloid cell
FSC-A on myeloid DC	23	0.76 (0.62-0.93)	0.008*	-0.28	0.11	cDC
FSC-A on monocyte	23	0.77 (0.62-0.97)	0.024*	-0.25	0.11	cDC
FSC-A on granulocyte	23	0.81 (0.67-0.99)	0.042*	-0.20	0.10	cDC
CD8 on CM CD8br	23	0.81 (0.65-1.00)	0.048*	-0.21	0.11	Maturation stages of T cell

## Discussion

Our research amalgamates extensive individual and collective GWAS data to methodically uncover how immune cells influence the emergence and progression of ADHD, considering genetics. The research offers indicative proof that immune cells may affect the risk of ADHD via a comprehensive genetic method, grounded in extensive GWAS summary data. By employing SNPs as key variables and combining various two-sample MR techniques, it was found that three immune cells: CD33 on Im MDSC, CD8^br^ NKT %T cell, and CD8^br^ NKT %lymphocyte are linked to the risk of ADHD. A number of immune cells could be linked to the emergence of ADHD.

The findings of our research indicate a heightened risk of ADHD correlating with a rise in the percentage of naive CD33 in Im MDSC. A novel group of immune cells, known as Myeloid-derived suppressor cells (MDSCs), have surfaced playing crucial roles in immune regulation (41). Within the mouse model, MDSCs are identified as cells that exhibit the myeloid cell lineage differentiation antigen Gr-1 and are further categorized based on the expression levels of the epitopes Ly-6G (42). The human phenotype manifests as LinHLADRCD33 or CD11bCD14CD33. In humans, they are distinguished by the standard immunophenotype of CD11bCD33HLA-DR++–/low and properties that modulate the immune system, resulting in reduced T-cell growth, stimulation of Tregs, obstruction of natural killer (NK) cell operations, and macrophage M2 polarization (43). The presence of CD33 diminishes in developed cells, evident in myeloid stem cells (CFU-GEMM, CFU-GM, CFU-G, E-BFU), as well as in myeloblasts, monoblasts, monocytes/macrophages, granulocyte precursors, and mast cells. This serves as an essential indicator and molecule in the study and recognition of MDSCs, referred to as Sialoadhesin (43). Neuroinflammation is intricately linked to the genetic makeup of CD33. Elevated levels of CD33 in the brain correlated with more pronounced cognitive deterioration (44). The escalation of neuroinflammation heightens the likelihood and accelerates the advancement of neurodegenerative and neurodevelopmental conditions, ADHD included, via various pathways like activation of glial cells, heightened oxidative stress, diminished neuronal activity, and alterations in neurodevelopment (45). Cytokines and chemokines that promote inflammation initiate the activation of adjacent stromal cells, leading to the release of glutamate (known as excitotoxicity), and heightening the permeability of the blood-brain barrier (BBB), thereby facilitating increased infiltration of immune cells into the brain tissue and intensifying the inflammatory reaction (45–47).

Natural Killer T (NKT) cells represent a crucial group within the unconventional T cell category. Dysfunctions and shortcomings in non-traditional T cells are linked to conditions like autoimmunity, persistent inflammation, and cancer (48). Lipid-based antigens, identified by NKT cells through the β2M-associated MHC class-I-like molecule CD1d, are categorized into two main types: type 1 and type 2 NKT cells. Type 1 NKT cells identify the standard lipid antigen of NKT cells, α-galactosylceramide (α-GalCer), and exhibit a CD1d-limited semi-invariant αβ TCR, consisting of a stable α-chain (Vα14–Jα18 in mice, Vα24–Jα18 in humans) linked to a restricted set of β-chains (Vβ8, Vβ7, and Vβ2 in mice, Vβ11 in humans)1,2 (49). It seems that humans possess a higher count of type 2 NKT cells, exhibiting a variety of TCRs that bestow extensive lipid antigen specificities1,3 (48, 50). Both CD8br NKT %T cells and CD8br NKT %lymphocytes belong to the category of NKT cells that exhibit the CD8 receptor. The category of CD8br NKT %lymphocytes is extensive, encompassing not just CD8br NKT %T cells but also various other lymphocyte groups that express CD8 and are part of the NKT cell group, with the brain being linked to the peripheral immune system via the lymphatic system (11). These cells are crucial in controlling immunity and altering inflammation, participate in diverse immune reactions, and are associated with the development of autoimmune disorders, viral infections, and cancer (48). CD8, a glycoprotein found on the cell surface, is vital for the immune system. This is mainly found on cytotoxic T cells, or CD8+ T cells, a specific group within T lymphocytes. CD8 serves as a secondary receptor to the T cell receptor (TCR) and engages with major histocompatibility complex class I (MHC-I) molecules on cells that present antigens (51). CD8 T cells evolve from naive to central memory and effector memory cells (16, 17). Studies indicate the impact of CD8 T cells on the functioning of neural progenitor cells (52). Earlier, CD8 cytotoxic T cells played a role in the development and operation of the brain (11). CD8 T cells within the central nervous system disrupt the balance of microglial and neuronal functions (11, 14). This type of infiltration may happen in persistent inflammatory conditions, like auto-immune and atopic diseases (15). The presence of CD8+ lymphocytes is indicative of the onset of autoimmune mental and neurological conditions in pediatric acute-onset neuropsychiatric syndrome (PANS), predominantly diagnosed with ADHD (52). Childhood attention problems are heavily influenced by the pro-inflammatory immune spectrum, where an increased count of Th1 and cytotoxic T cells correlates with elevated attention problem scores, independent of simultaneous psychopathological conditions (53). Among all CD8 T cells, an elevation of 1SD in either naive or central memory cells correlated with an increase of 6.9% (95%CI: 2.0~12.1) and 6.4% (95%CI: 1.5~11.6) in scores for attention problems (53). The function of NKT cells in enhancing CD8 T-cell reactions and memory development in immunization, pathogenic infections, and tumor immunity in both mice and humans has been extensively studied (54, 55). NKT cells uniquely contribute to enhancing CD8 T-cell priming and secondary reactions, or they control the destiny of CD8 T-cells (be it death or memory formation) through their interaction timing, location, and specific NKT cell groups. Furthermore, age-related alterations in the quantity and roles of NKT cells are notable, with scant studies indicating that these cells hinder T-cell immune responses (56).

In investigating the developmental impact of ADHD on the immune mechanisms of the human body, although multiple adjustments have been made through repeated examinations without reaching association at the FDR level, 20 immune cells exhibit a causal effect at a nominal significance level. Among these, 85% of immune cells show decreased expression, implying that ADHD may lead to a decline in the body’s immune levels. Chen Ziling et al. (57) first reported a correlation between ADHD and recurrent upper respiratory tract infections. The results indicate that the incidence of recurrent upper respiratory tract infections in children with ADHD is 1.769 times higher than in non-ADHD children. In a clinical study involving 60 children with ADHD and 50 normal control children (58), it was found that the activity of CD3+, CD4+, and CD4+/CD8+ T lymphocytes in the ADHD group was lower compared to the normal control group. This is consistent with our research, indicating a decrease in CD3 cell expression levels. However, other studies have proposed contrasting conclusions: Children with ADHD exhibit higher levels of CD3, CD4, and CD25 Foxp3 (Tregs) compared to the healthy control group (13).

Collectively, our findings suggest the immune system’s response influences the emergence of ADHD. This plays a crucial supporting role in clinically assessing disease prognosis and treatment and also guides the creation of novel medications. Nonetheless, the development of ADHD is intricate, the diverse clinical characteristics of various immune cells implicated in ADHD are evident, and typically, a solitary treatment fails to yield favorable outcomes. Consequently, a deeper exploration is needed into how innate immune cells interact with adaptive immune cells in ADHD. There are certain constraints in our research. Initially, our choice was GWAS summary datasets, focusing on the most extensive sample sizes for immune traits and ADHD. Nonetheless, the data on immune cells and ADHD originated from varied studies, exhibiting discrepancies in sample sizes, quality control techniques, and ethnic backgrounds. The most direct approach to mitigate bias in population stratification is to include cohorts with identical genetic profiles in genetic association studies. However, the limited effectiveness of traditional GWAS statistical tests has prompted a trend towards expanding sample sizes for multicenter GWAS. Since all the GWAS included in this study were conducted in European populations, the potential bias in population stratification was relatively minor. Despite Sardinians being part of the European genetic lineage, akin to Danish and Icelandic, this group exhibits a degree of diversity. Addressing racial diversity issues in two-sample MR remains an unresolved challenge. The surge in cross-ethnic research necessitates the development of innovative statistical techniques to connect studies involving various races. As an illustration, a study conducted a GWAS meta-analysis in collaboration between the UK Biobank and FinnGen GWAS, without any specific procedures (59). Furthermore, a variety of European GWAS studies were carried out, encompassing diverse European ethnic groups (60). Consequently, we stressed the importance of carefully interpreting the results yielded in this research. Despite meticulously choosing IVs to meet different model criteria and conducting thorough sensitivity studies to minimize potential confounding variables, our analysis relied on summarized datasets, lacking individual data. As a result, further studies on population stratification are needed, particularly those investigating the impact of disease severity on individual SNPs, while also considering factors such as sex and age. These additional studies are necessary due to the limitations preventing the exploration of desired traits in the current research. Thirdly, despite the application of multiple FDR adjustments, a lenient criterion for selecting SNPs (owing to a small sample size) could lead to some level of incorrect positive results. To sum up, our findings might offer new perspectives on the immune response to the emergence of ADHD, necessitating additional experimental studies to delve deeper into the connection between recognized immune characteristics and the risk of ADHD.

## Data availability statement

Information can be accessed through a publicly accessible, open-access database. URLs for data: Accessible for downloading GWAS summary statistics on 731 immune characteristics from the GWAS Catalog (Study ID: GCST90001001 ~ GCST90002000, https://www.ebi.ac.uk/gwas/home); ADHD summary statistics for GWAS can be obtained from https://www.med.unc.edu/pgc/download-results/. Every code employed in the study can be obtained from the respective authors.

## Ethics statement

Ethical approval was not required for the study involving humans in accordance with the local legislation and institutional requirements. Written informed consent to participate in this study was not required from the participants or the participants’ legal guardians/next of kin in accordance with the national legislation and the institutional requirements. Written informed consent was obtained from the individual(s) for the publication of any potentially identifiable images or data included in this article.

## Author contributions

JH: Writing – original draft. D-FC: Writing – review & editing. F-FL: Data curation, Writing – review & editing. K-PY: Methodology, Writing – review & editing. J-YX: Software, Writing – review & editing. H-TZ: Writing – review & editing. X-BX: Writing – review & editing. JC: Writing – original draft, Writing – review & editing.
